# New Generation of MOF-Monoliths Based on Metal Foams

**DOI:** 10.3390/molecules27061968

**Published:** 2022-03-18

**Authors:** José J. Delgado-Marín, Dennis P. Izan, Miguel Molina-Sabio, Enrique V. Ramos-Fernandez, Javier Narciso

**Affiliations:** Laboratorio de Materiales Avanzados, Departamento de Química Inorgánica-Instituto Universitario de Materiales, Universidad de Alicante, Ctra. San Vicente-Alicante s/n, E-03690 San Vicente del Raspeig, Spain; jj.delgado@ua.es (J.J.D.-M.); paul191994@gmail.com (D.P.I.); m.molina@ua.es (M.M.-S.); narciso@ua.es (J.N.)

**Keywords:** monoliths, Metal Organic Frameworks, Zamak5

## Abstract

Herein, it has been developed a method to prepare metallic foams starting from Zamak5 (ZnAlCu alloy) with different pore sizes. The Zamak5 metallic foam is designed to serve as a support and metallic precursor of ZIF-8. In this way, composite materials MOF-metal can be prepared, these composites have a large number of application in energy exchange processe such as: adsorption or chemical reactions. Additionally, this method of sythesizing MOFs is environmentally friendly thanks to absence of solvents. Hanerssing the low melting point of the linker, the linker is infiltrated into the foam where the foam and the linker react to form the ZIF-8. In this way we have managed to transform part of the foam into ZIF-8 crystals that remain adhered to the foam. The foams have been characterized and modeled studying the mechanical and electrical properties, finding that both can be predected by various models. Among these, Ashby and Mortensen models for mechanical properties and Ashby and Percolation model for electrical properties stand.

## 1. Introduction

Metal Organic Frameworks are coordination polymers that have a large number of applications, such as: catalysis [1,2], gas adsorption [3,4,5,6] and separation, sensors [5], drug delivery [7,8,9] among others. These myriad of applications can be obtained thanks to their hybrid nature so, it is possible to modulate both textural and chemical features. Many MOFs have shown their great effectiveness at the laboratory level for a given application. A good example of this are MOFs used for adsorption, which overpass the adsorption capacity of their competitors (activated carbon, zeolites etc.); however, cannot be industrially applied. The main problems is the lack of stability and the high cost of manufacturing due to the use of organic solvents (which, of course, are not very environmentally friendly). However, currently there are two families of MOFs, Zeolitic imidazolate frameworks or ZIF (maximum representative is the ZIF-8) [10,11,12,13,14,15,16], and the Universitetet i Oslo, UiO (maximum representative is the UiO-66) [17,18,19,20,21,22,23,24,25], which are very stable and their production cost is not excessive.

Apart for being effective for catalysis or adsorption, a number of additional properties are mandatory to be applied in both processes. It is necessary to shape the MOF as pellets or monoliths (preferably the latter) to avoid pressure drop (in both reactor or adsorber), and favors the heat and mass transport. The shaped MOFs must have good thermal and electrical conductivity due to two reasons: because in a chemical reaction or in an adsorption process there is an energy exchange and, if electrical conductivity is present, it is easy to modulate the energy exchange through the Joule [26] or Peltier effects [27].

Although this problem is well known, research into the development of MOFs monolith having high thermal and electrical conductivity has not been deeply developed, even though, we can highlight four many research lines trying tackle this problem. (i) The preparation of MOFs-Polymeric Beads composites: the original idea has been patented [28] and consists of preparing composite beads containing the powder of MOFs and a polymeric binder. They demonstrate that the MOF’s porosity is accessible in the final material and with this approach, insulator MOFs monolith are achieved. (ii) The preparation MOFs monolith by extrusion method based on the knowledge developed for 3-way catalysts technology. Different systems have been developed, for example for the Cu_3_(btc)_2_ [29] by using clays such as attapulgite, montmorillonite, kaolin, and bentonite as binder, finding that the surface area obtained is acceptable as well as the mechanical properties (again not electrical or thermal conductivity is present). (iii) Monolithic MOF-gels, the idea is to use the sol-gel technique instead of binder to generate monoliths; however, the use of surfactants modifies the procedure in order to crystallize MOFs, which involves more complex processes. A review article has recently appeared [30,31], where the latest advances in this way of producing monoliths are explained in detail. (iv) The last method to shape MOF is the growing of thin layer of MOF on structured supports, for example, Ramos-Fernandez et al. [32] published a method to growth MIL-101(Cr) crystals on cordierite monoliths. This approach has the advantage that MOF and monolith synthesis is decouple. It suggests that the growing of MOF on the surface of a monolith can give many degrees of freedom to obtain the desired final material, since different support and MOFs can be used. The principal disadvantage is that the final composite material might have a low amount of MOF, but this scenario can be preferable for several applications.

In this contribution, it first prepared metal foams as a support of MOFs, and later, the MOF is grown on the foam using the own metal foam as metal source of the MOF.

**Figure 1:**
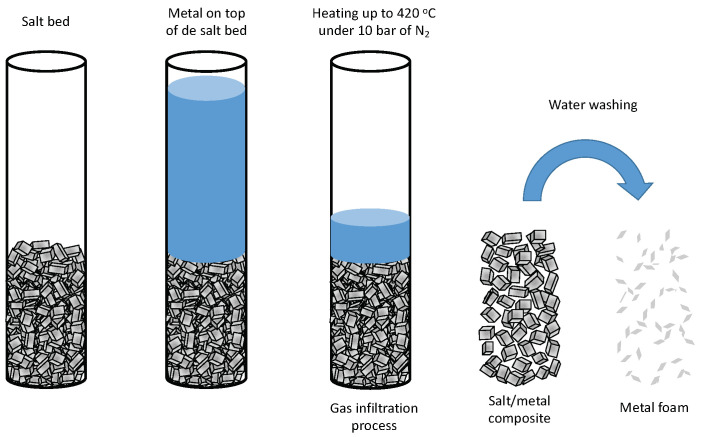
Experimental procedure to manufacture metal foams by Gas Pressure Infiltration (GPI).

The preparation of the metal foam consists of several steps: (i) a bed of NaCl crystal is prepared by gently compacting NaCl crystals in a tube (see Figure 1), (ii) on top of the salt bed, an amount of the desired metal is placed, (iii) then, the tube is heated until the temperature of melting point of the metal is reached while an exogenous pressure is applied. In this process, the liquid metal is infiltrated in the salt bed in a so-called gas pressure infiltration (GPI), (iv) once the system is cool down, the metal/salt composite is immersed in water to easily dissolve the NaCl producing a connected metal foam that can have between 40 and 1000 microns depending on the particles size distribution of the salt crystals.

The obtained metal foam is used as support and precursor of the MOF. Zamak is a Zn alloys that is deeply used in manufacturing zippers, buttons, rivets or another clothing accessories. These alloys are used for such as purposes mainly because the melting point of this range from 400 to 500 °C, depending on the composition. The low melting point of the alloys allows the production of metal pieces by casting in mild conditions. Furthermore, the surface tension of the liquid Zamak is 750 mJm^−1^, this low value make that liquid Zamak wets almost of kind of surfaces favoring the preparation of pieces in small casts [33,34]. Considering these two features of Zamak, we decided to use this alloy as playground to prepare metal foams that would be later transform into a MOF [35].

Recently, we have developed a method to partially convert the Zn metal alloys into ZIF-8 by immersing the metal alloys into melted 2-methylimidazole. Thus, the targeted MOF would be ZIF-8 [36].

## 2. Experimental

NaCl particles of analytical grade (purity > 99.5 wt%) from Sigma-Aldrich (Burlington, MA, USA) has been used. These particles have a very wide particle size distribution (40–500 microns), subsequently sieving was performed to obtain three different lots with a narrow size distribution (150, 270, 420 micros). Zamak5 (Al 4%, Cu 1%, Zn 95%) has been used for infiltration (for a deep characterization see the reference [36]). 2-methyl-imidazole and acetone from Sigma-Aldrich has been used without further purification.

The replication method has been previously used for the authors [37] to obtain cellular materials. This method consists of the following steps (see Figure 1 and Figure S1), (a) NaCl is packaged in the tubular crucible (20 mm internal diameter), (b) the metal is introduced on the top and it shows into the tubular crucible in the chamber, applying moderate vacuum and heat until 420 °C, (c) increase of exogenous pressure (1 MPa, N_2_) during 5 min, and after, the system is cooled down (is mandatory to use a large excess of metal to have a top layer of metal in the bed, to ensure that all the impurities and defects go to the free metal), (d) the excess of metal is removed by cutting the top layer metal, (e) the Zamak/NaCl composites is immersed in water during 24 h to dissolve the salt and obtain the foam.

The partial transformation of foams into ZIF-8 has been obtained following the next procedure: one disk of Zamak5 foams (5 mm of height) were place in a vial together with 2.5 g of 2-methyl-imidazole (2-mIM). After that, the vial was flushed with N_2_ and sealed with a silicone cap. The sealed vial was placed in a pre-heated oven at 160 °C, and kept in the oven for 72 h. At the end, the vial was broken and a solid was obtained. The solid was composed of Zamak/ZIF-8 composite and some impurities (i.e., unreacted linker) so, it needed to be purified. It done by Soxhlet extraction for 24 h and using acetone as the solvent.

The morphology of the materials was examined by scanning electron microscopy, SEM. The analysis was performed on gold-coated samples by using a S3000N microscope (Hitachi). X-ray diffraction (XRD) analysis was carried out with a Bruker D8-Advance diffractometer (Billerica, MA, USA) (operating at 40 kV and 40 mA), using a CuKα radiation (λ = 1.54056 Å). The samples were scanned from 2.5° to 80.0° (2θ), at a scanning rate of 1.0°·min^−1^. The compression strength of the different disk foams was measured using a universal testing machine (Instron 4411) with a 0.1 mm min−1 crosshead speed. Prior to the compression tests, Zamak (99.999 wt%) compression modulus was measured under the same testing conditions. The flexural modulus obtained for Zamak5 was 95 GPa, fully consistent with literature. Measurements of electrical conductivity were carried out with an eddy-current apparatus (Sigmatest 2.069, equipment fabricated by Foerster Instruments Inc., Pittsburgh, PA, USA).

## 3. Results

### Manufacture and Characterization of Foam

The infiltration process is not so straightforward and, in order to obtain high quality salt/metal composite materials (without closed porosity or trapped oxides) using GPI, we must consider 3 factors: the threshold pressure (minimum pressure necessary for the infiltration of the liquid into the salt bed), the Reynolds number, and the capillary number, both of them related to the shape of the front flow of the liquid metal through the salt bed or preform [38].

For the calculation of the threshold pressure, it can use the following expression

$$P_{0}=S_{0}\gamma_{lv}cos\theta$$

where *S*_0_ is the particle surface area per unit of volume of liquid matrix, *γ_lv_* is liquid vapor surface tension and *θ* is the contact angle between the liquid metal [39,40]. This expression is derived from the Young-Laplace equation, that describes the capillary pressure in a cylindrical pore. In this case *S*_0_ is used instead of the inversed of the capillary diameter, since, *S*_0_ represent the mean average size of the pore in the salt bed [40].

The contact angle in these conditions usually ranges between 125 and 150°, due to the metal is covered with a layer of oxide so the melted salt actually sees its oxide. The surface tension is approximately 750 mJ·m^−1^ [33,34,41,42], the main problem is to measure the surface area of the particles, since their B.E.T surface area is less than 0.5 m^2^/g, the usual method to assess the surface area is the adsorption of N_2_ at 77 K, it cannot be used, since we are below detection limit. Therefore, we can make an approximation, which is to determine the surface area using the particle size distribution curve (Figure S2), and assume that the particles are cubic (typical shape of the NaCl cristallites). Taking into account these data, we obtain that the threshold pressure is 0.18, 0.11 and 0.07 MPa for the different NaCl particle sizes. As the applied infiltration pressure is 1 MPa, the infiltration takes place, also as we know that if we apply 3 times the threshold pressure more than 99% of the interparticular space is infiltrated, and we would have a perfect continuous system, and it has been demonstrated for others metals [37,40,43].

As it has commented above, we must ensure that the infiltration front is flat to avoid entrapment of surface oxides or the generation of void not accessible. The first conditions to have a flat front is to achieved a laminar flow, since turbulent flow will produce both undesired effects. In chemical engineering the most common way to determine what type of flow is reached, laminar or turbulent, is the use of the dimensionless Reynolds number that must have a value below 2000 to be sure that the laminar flow is achieved [39]. The Reynolds number (Re) is defined as follows:

Re=ρvDη

where *ρ* is the density of the liquid metal, *D* is the diameter of the channel, which is the interparticle distance that can be assumed to be about 1/3 of the average size of the particles, *ν* is the fluid velocity, in our equipment the maximum velocity that can be achieved is 5 mm/s [40]. Thus, the maximum Reynolds number would be 4, which would correspond to bed made with the largest NaCl particles, that has the largest diameter of the channel so the maximum possible Reynolds. This implies that the infiltration process is in a laminar regime. The fact that the laminar flow is achieved does not mean that we obtain a flat infiltration front, because that is just the first condition. It is necessary to know if there is fingering or not (where de front flow is not flat), the capillary number must be less than 1. The capillary number (Ca) is defined as:

$$\mathrm{Ca}=\frac{\eta v}{\gamma_{lv}}$$

The capillary number is the relationship between viscous drag forces versus surface tension [40]. Substituting the values, we obtain that the capillary number will oscillate between 10^−4^ and 10^−5^. Therefore, all the conditions are met to produce a flat infiltration front. Three salt beds with different particles size distribution were prepared and infiltrated in all cases. The Re, Ca and the *P*_0_ met the conditions to get an appropriated infiltration. Actually, the three salt/metal composites were successfully obtained. After washing the composites, the metal foams are obtained. In order to check the quality of the foams, density, electrical conductivity and mechanical properties have been measured and analyzed.

If the density of the foam is equal to the density of the bulk metal indicates that the foams are continuous and all the porosity is accessible and there are not voids or oxides entrapped in the walls of the foam. The way to measure this density is using He pycnometry. As it can be seem in Table 1 density of the foam is almost equal to the density of the bulk Zamak (6.8 g/cm^3^) indicating the high quality of the foams. Once is known that the three Zamak foams are solid an there aren’t oxides entrapped or inaccessible voids, we can calculate structural parameters of the foams; porosity or the width and the length of the ligament between the pores.

The porosity is calculated by measuring the size and the weight of the foams pieces and calculating the density, and comparing this density against the measured with He picnometry. It gives the percentage of empty space on the foams. The values of the porosity range from ~69 to 72% of porosity. These values are very commonly found in the field of metals foams and are the expected [35,44]. The length (*l*) and width (*t*) of the ligament is calculated (see theoretical background in supporting information). Table 1 shows in the column x the ration *t*/*l* of the different foams. In order to compare these theoretical values with the measured ones, we observed our foams with scanning electron microscopy (SEM) (shown in supporting info). The values for the *t*/*l* calculated and measured by SEM range from 0.174 and 0.185 and are practically the same. So, the method to estimate the *t*/*l* values match with values observed by SEM. With these results in hand, it can be said that three high quality metal foams have been synthetized.

Regarding the mechanical properties, several compression tests have been carried out on the foams to determine the elastic capacity using the Young’s modulus (*E*).

Once of the most common use of metallic foams is the energy absorption in an impact event and this fact has generated a very extensive study in the plastic properties of these materials, however, some authors have carried out other types of micromechanical analysis of these materials. Within the micromechanical studies, two can be highlighted: those of Professor Ashby and those of Professor Mortensen. The studies made by Professor Ashby [45], who makes an exhaustive study for different types of open foams, modelized the mechanical properties (Young’s module) of the foams with respect to the porosity (*t*/*l* ratio). The Young’s modulus defined form Ashby model for the case of square regular foams is as follow:

$$E=E_{0}{\left(\frac{t}{l}\right)}^{3}$$

where *t* is the width and *l* is the length of the ligament, respectively, as shown in Figure S4. *E* is the measured Young’s modulus of the foams. *E*_0_ is the Young’ modulus of the bulk Zamak5. Actually, in a real foam obtained by this method, the size of the ligament and the length cannot be measured, but we can do an approximation and it has been assumed that our system is regular as shown in the Figure S5. Based on the previous parameters it can be defined that relative density of the foam must be between two values: 0.35 and 0.25. Thus, we could obtain a relationship between the foam density and the parameter, as shown in Table 1, and the theoretical properties that would be obtained.

Another empirical model is the one developed by Mortensen [38] for foam manufactured in the same way, in which the foam density is directly related to Young’s modulus.

**Figure 2:**
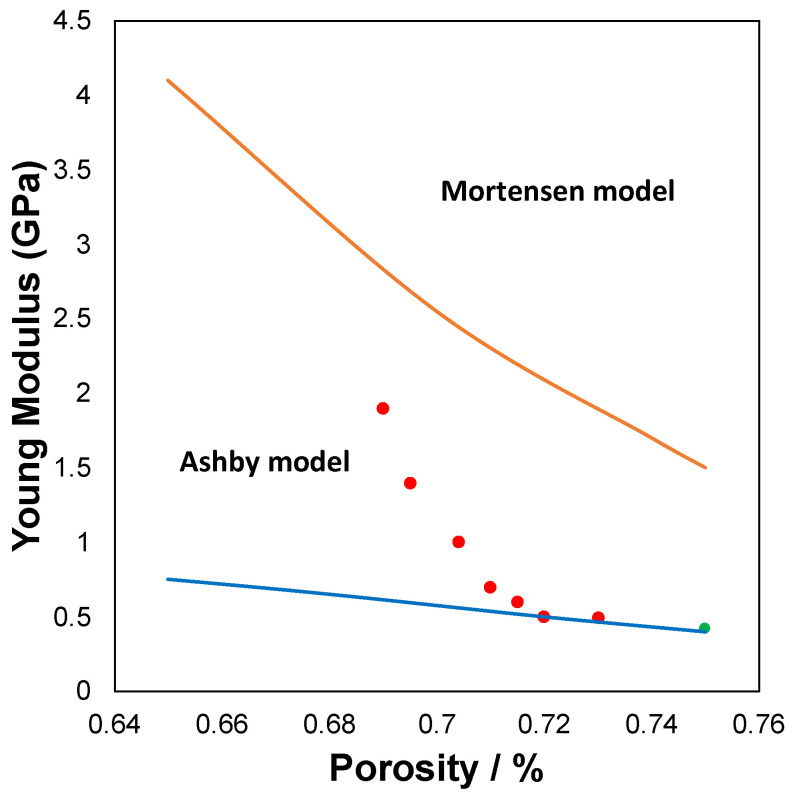
Evolution of Young Modulus at function of the Porosity. Red point are the experimental data. Circles are the experiment points: red are the foam and green the composite.

Figure 2 shows plot the Young modulus versus foam porosity. The continuous lines represent the theoretical models, both Ashby and Mortensen. The dots are the experimental values and these are lying between the two models. It is important to note that when the porosity increases by 70%, the experimental data practically fit the model proposed by Ashby, which indicates that the foam is open regular.

Two approaches are used to model the electrical conductivity in this types of foams [46]. A well-known one was the approach developed by Maxwell 150 years ago, which is based on introducing a non-conductive particle into a conductive material, where the analytical expression is as follows:

$$\sigma =\sigma_{0}\frac{2\left(1-P\right)}{2+P}$$

**Figure 3:**
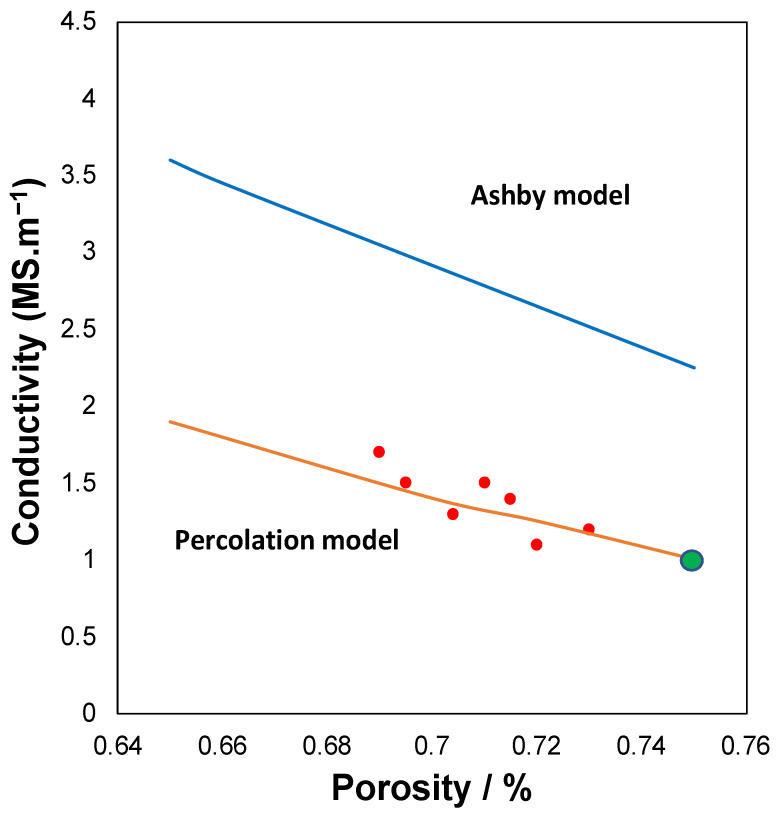
Evolution of electrical conductivity at function of the porosity. Circles are the experiment points: red are the foam and green the composite.

That would serve as an upper limit. The other possibility is to suppose that there is percolation, whose expression is as follows:

$$\sigma =\sigma_{0}{\left(P_{\;c}-P\right)}^{m}\;$$

where *P_c_* is the critical porosity or percolation limit (property is equal to zero), and m range between 1.3 and 3 [47]. The main problem is to know the percolation limit for these structures. There are other more complex multiparameter models that are semi-empirical, and whose structure of the equation closely resembles the percolation theory [47]. But, as with the mechanical properties, Professor Ashby [45,48] has developed models for the cellular structures discussed in the mechanical properties, obtaining the following expression:

σ=σ0(α(1−P)+(1−α)(1−P)32) 

where it takes two possible values of 0.33 for closed foams and 0.05 for open foams. The electrical conductivity data are shown in Table 1, and are represented together with the models previously proposed in Figure 3. The principal difference observed is that, in this case, the Ashby model does not fit the experimental data, but rather the percolation model, is the one that best fits our results. The fact that there is a great infinity of models due to the great dispersion of the data found, but what is usually consistent is the evolution with porosity for the same group of foams (manufactured with the same technique, and by the same authors). Also, the percolation limit is so high (*P_c_* = 0.95). it is also indicative that connectivity is almost total, as previously predicted. For a perfect connectivity of the foams Pc should be 1.

In conclusion, a series of foams with different pore sizes with open porosity, metallic conductivity ranging from 1.1 to 1.7 MSm^−1^ (10% of the bulk Zamak5) and mechanical properties have been synthesized within the expected values for these types of solids. The fact that the both mechanical properties and electrical conductivity can be modeled means that we can predict properties for unprepared foams.

Once it has been demonstrated that a several high-quality foams can be prepared, these foams were used as precursor and substrate of ZIF-8. For doing so, it was used a method we have recently developed. The details of synthesis method are reported elsewhere [36]. This method consists of immersing the metallic foam in melted linker (2-methylimidazole). The melting point of the linker is 165 °C. The reaction between the Zn of the metal foam and the linker happens at this temperature, and part of the metal foams is transformed into ZIF-8. In our previous article [36], the synthesis was carried out both on a flat plate and in powder where it was possible to successfully verify the synthesis of ZIF-8 from Zamak5 and the melted linker. The problem with making the synthesis on the foam is that it is a necessary condition that the linker wet the Zamak5 (contact angle must be lower than 5), then infiltration occurs and it reacts with the metallic foam. The contact angle of the linker on the Zamak5 has been measured and it has been found that the contact angle is less than 5 degrees which is the detection limit of the equipment; in addition, this occurs in less than 5 s, so we can say that the thermodynamic contact angle is close to zero, so we can use the reduced Darcy equation to estimate the infiltration time: [40].

$$h^{2}=\frac{2kt}{\mu \phi }\;P\;$$

where *k* is the permeability and *μ* is the viscosity, *P* in this case would be the threshold pressure, *φ* is the porosity, and h is the infiltrated height. As in our case the preform has a height of 0.5 cm, the permeability of this type of preform comprises a range between 10–12 and 10–14. The viscosity is in the order of mPas, and the threshold pressure ranges between 10^−1^ and 10^−2^ MPa as mentioned above, so it can calculate the estimated infiltration time that ranges between a few seconds and a few minutes, which is between 1 and two orders of magnitude lower than the reaction time we have used.

**Figure 4:**
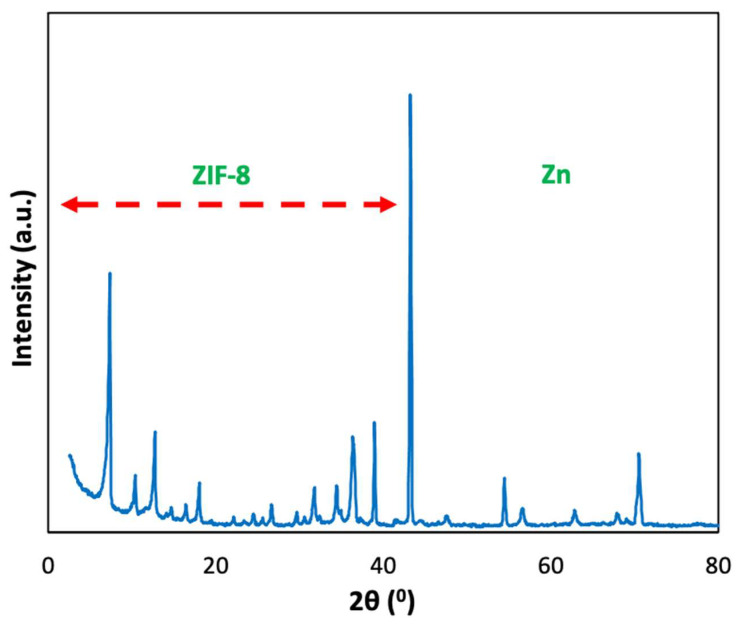
X-ray diffraction pattern of the composite ZIF-8/Zn.

In order to check the success of the synthesis of the ZIF-8, we have performed XRD diffraction. The results are shown in Figure 4, as it can be seen, the peaks pattern ZIF-8/metal composites perfectly match with the pattern of ZIF-8. Furthermore, some additional peaks at 2 theta higher that 35 are also present in the composites. These peaks are attributed to the Zamak that has not been converted into ZIF-8. This technique proves the success of our approach of converting Zamak foams in ZIF-8/Zamak composites.

**Figure 5:**
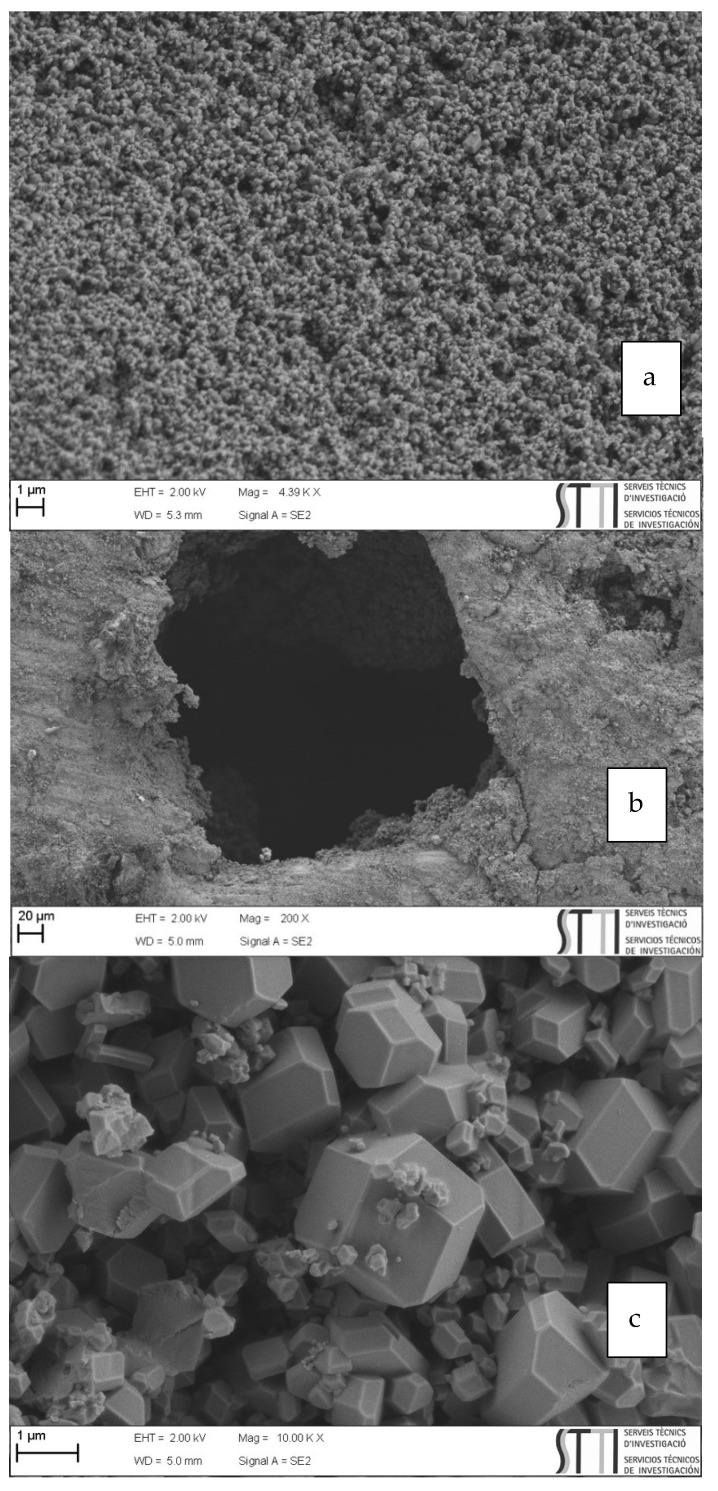
Shows different micrographs of the composite material obtained. First (**a**) Zamak5 is covered by the ZIF-8 where it is clear that the pore is cubic as discussed above. The other two micrographs (**b**,**c**) show that the material is fully coated and the microcrystals have the typical cubo-octahedral topology of ZIF-8.

We have further analyzed the images and we have calculated the crystal size distribution by measuring more than 1000 crystal at the SEM images (Figure 5). The results have been included at the supporting information. As it can be seen, the crystal size distribution is very broad and centers at 250 nm size.

## 4. Conclusions

The manuscript shows that the production of Zamak5 foams with the GPI technique on a NaCl bed is feasible and that it allows us to obtain foams with perfectly controlled pore size. The mechanical and conductive properties evolve according to the Ashby and percolation models, so the properties of the ZIF-8/Zamak5 composites can be predicted.

The fabrication of the ZIF-8/Zamak5 composites have been successfully carried out obtaining a ZIF-8/Zamak monolith having electrical conductivity.

## Figures and Tables

**Table 1:** Physical properties of the manufactured composites.

Sample	He Density (g/cm^3^)	Porosity (%)	*t*/*l*	*E* (GPa)	*σ* (MSm^−1^)
150 mm	6.75 6.78	0.69 0.71	0.185 0.178	1.9 0.7	1.7 1.4
270 mm	6.77 6.81	0.695 0.73	0.183 0.172	1.4 0.46	1.4 1.2
420 mm	6.79 6.78 6.80	0.705 0.715 0.72	0.179 0.175 0.174	0.962 0.586 0.501	1.2 1.3 1.1

## Data Availability

The data presented in this study are available in article.

## Supplementary Materials

The following supporting information can be downloaded at: https://www.mdpi.com/article/10.3390/molecules27061968/s1, In the SI is included experimental and theoretical details as well as additional characterization of the prepared materials. References [49,50,51,52,53,54,55,56] are cited in Supplementary Materials.

## Sample Availability

Not available.
