# Supplementary material for: New Generation of MOF-Monoliths Based on Metal Foams
Source: Molecules. 2022 Mar 18;27(6):1968. doi: 10.3390/molecules27061968 (PMC8955634; doi:10.3390/molecules27061968)
Supplement: Supplementary file 1 [file molecules-27-01968-s001.zip › molecules-1614136-supplementary.pdf]

New generation of MOFs-monolith based on metal foams

José J. Delgado-Marin,[a] Dennis P. Izan[a], Miguel Molina-Sabio[a], Enrique V. Ramos-Fernandez[a*], Javier Narciso[a]

Experimental details,

The device used to perform the infiltrations has been designed and manufactured by the authors and previously described [49,50]. For the experiments carried out in this paper, the configuration shown in figure S1 has been used. It shows that it is possible to perform multiple experiments at the same time, we have used the configuration of 6 experiments, if we need larger foams we could use the configuration of 3 experiments.

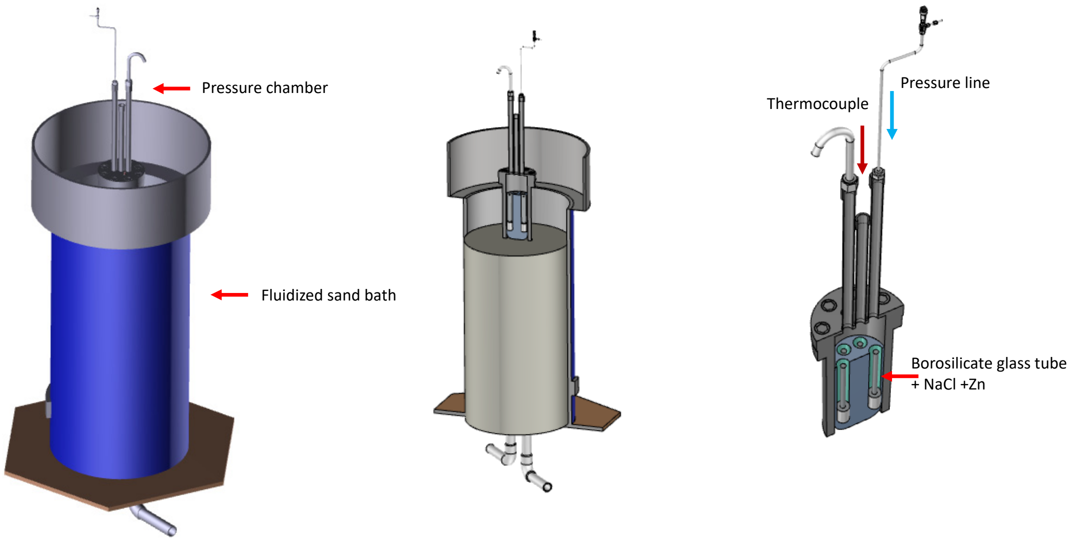

Figure S1. *Device for manufacture metal foam.*

The NaCl particles have been sieved into three fractions, eliminating particles greater than 500 microns and less than 100 microns. Particle size distribution of the 3 fractions was obtained using a Coulter laser particle analyzer. The distribution is shown in Figure S2. The distributions are centered at 150, 250 and 400 microns approximately, it is clearly appreciated that the smallest particles present a wider distribution.

Contact angle has been done following the procedure describe in [51]. Contact angles were measured with a Rami-Hart 100 goniometer, coupled with a hot chamber at 150 ºC. Single drops (2 microliter) of 2methylimidazol, were placed on the Zamak-5. Average values of at least three drops. To have higher accurate in the contact angle measurements has been done the profile analysis describe in [52].

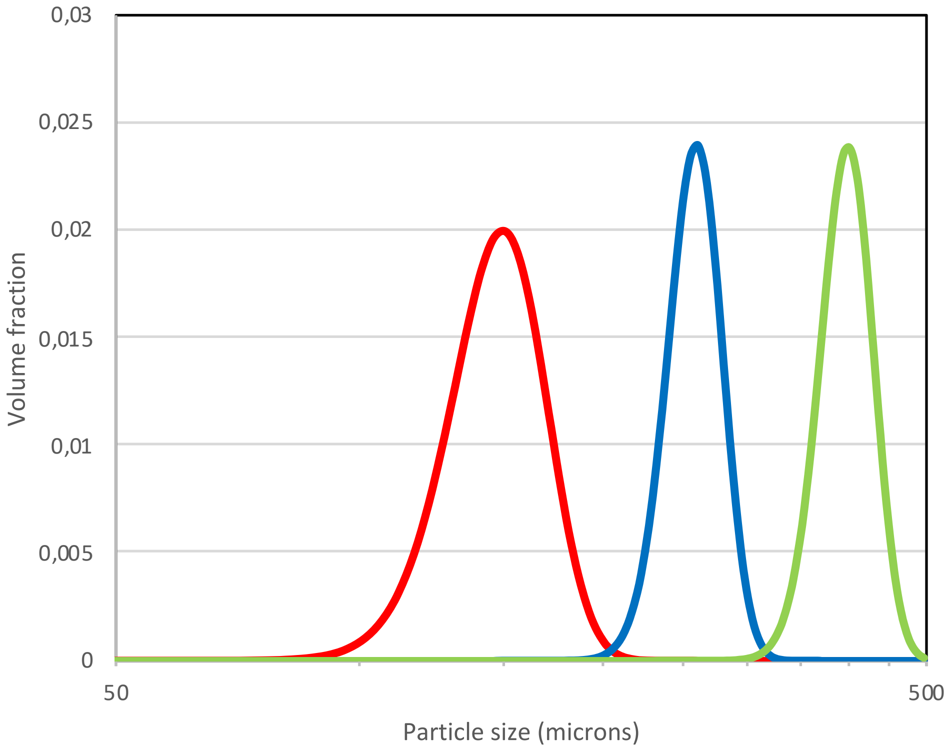

Figure S2. Particle size distribution of the three NaCl fraction obtained by sieving of commercial NaCl.

Theoretical background.

The modeling of mechanical properties in cellular materials is quite complex, especially those obtained by the replication process where we are in an intermediate situation. For low porosity (<15%) there are different models, from Griffith's original for brittle materials to the most advanced ones such as the MSA (Minimun Surface Area) [53] which is widely used, where a fairly rigorous micromechanical analysis has been made, especially for the shape of the pore (ie b = 5 sphere, b = 8 elongated)

$$E = E_0 \exp\,(-bP)$$

At the other extreme is the micromechanical analysis by Professor Ashby and his group on open-structure cellular materials [54], which normally move between 75 and 95% porosity, in addition the structures are regular and introduces the term ligament, which can be observed in the Figure S3.

This research group has carried out a large number of calculations for different types of cells. Figure S4 shows a cubic cell, which in principle would be the most similar to our foams since the NaCl particles are cubic. Note that for such a unit cell having a t / l ratio of 0.1, the porosity would be 90%. Specifically for said cell the relationship between the Young's modulus of the material and that of the foam obtain the following expression

$$E = E_0 ({}^{t}/_{l})^3$$

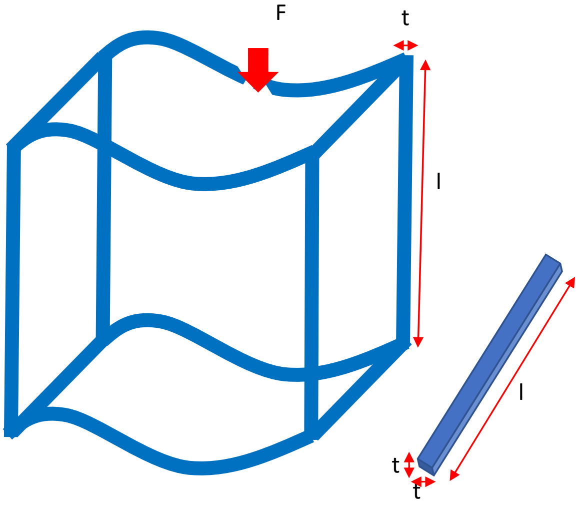

Figure S3. *Scheme of the modeling proposed by Ashby et al. For the calculation of the mechanical properties (elastic zone) of cellular materials. Where the parameters used is the relationship between the length (l) and the width (t) of the ligaments, which it considers to be square section bars.*

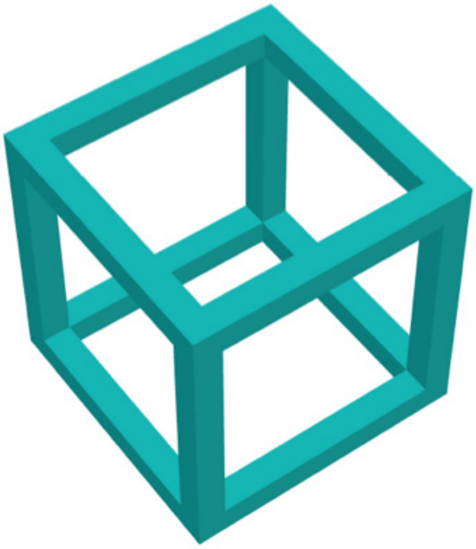

Figure S4. *Modelized structure for Zamak-5 foam obtained by replication process based of the model proposed by Ashby et al.*

We can also find semi-empirical models in the literature, one of the best known being those of Prof. Mortensen [55,56], where he relates the evolution of elastic properties with the evolution of the porosity, as shown in the following equation.

$$E = 0.6E_0(1 - P)^{2.7}$$

Just note that this model is valid for traditional aluminum foams obtained through the replication process.

The relationship of t / l with porosity can be obtained from the geometric considerations of the figure S4 and it would be the following equation, assuming that l is equal to 1:

$$P = 1 - [4t^2 + 8t^2(1 - 2t)]$$

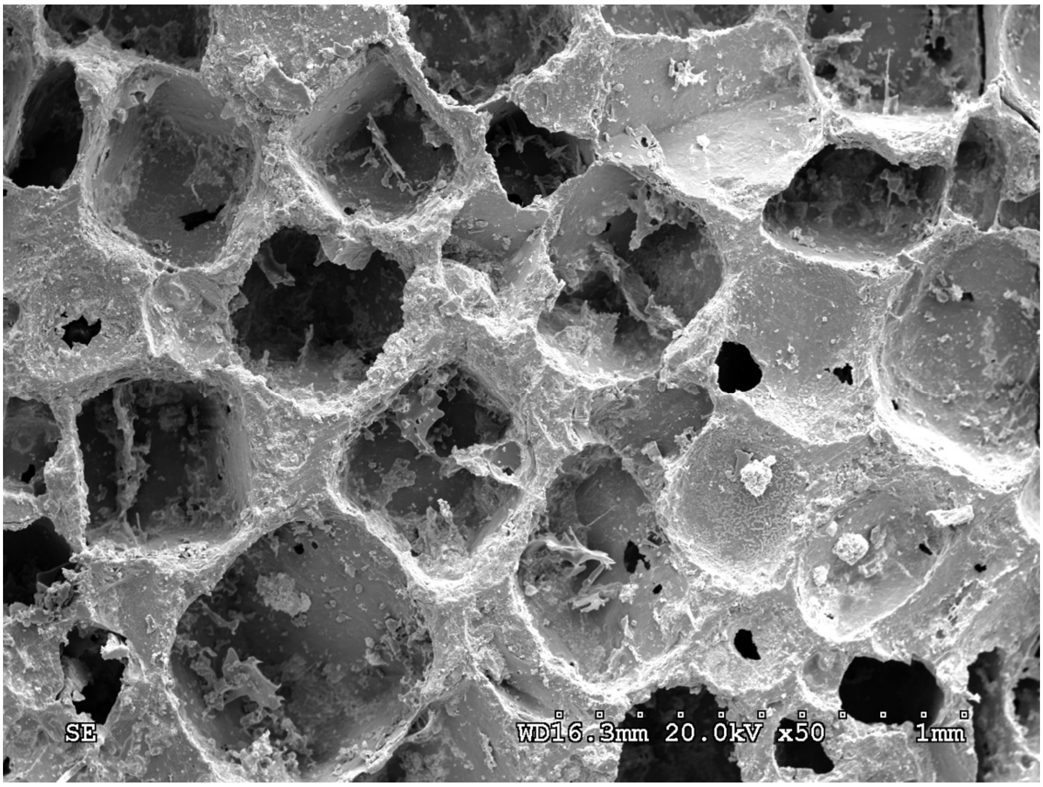

Figure S5. Micrograph of the foam.

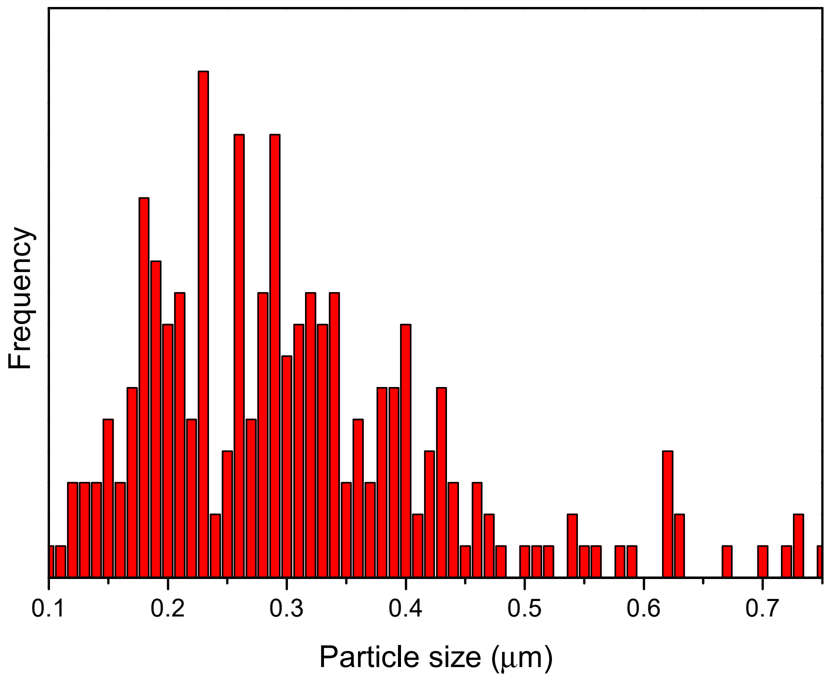

Figure S6. Crystal size distribution calculated by analyzing the SEM imagenes.
